# CRISPRlnc: a machine learning method for lncRNA-specific single-guide RNA design of CRISPR/Cas9 system

**DOI:** 10.1093/bib/bbae066

**Published:** 2024-02-29

**Authors:** Zitian Yang, Zexin Zhang, Jing Li, Wen Chen, Changning Liu

**Affiliations:** CAS Key Laboratory of Tropical Plant Resources and Sustainable Use, Yunnan Key Laboratory of Crop Wild Relatives Omics, Xishuangbanna Tropical Botanical Garden, Chinese Academy of Sciences, Kunming 650223, China; College of Life Sciences, University of Chinese Academy of Sciences, Beijing 100049, China; CAS Key Laboratory of Tropical Plant Resources and Sustainable Use, Yunnan Key Laboratory of Crop Wild Relatives Omics, Xishuangbanna Tropical Botanical Garden, Chinese Academy of Sciences, Kunming 650223, China; CAS Key Laboratory of Tropical Plant Resources and Sustainable Use, Yunnan Key Laboratory of Crop Wild Relatives Omics, Xishuangbanna Tropical Botanical Garden, Chinese Academy of Sciences, Kunming 650223, China; Hunan Provincial Key Laboratory of Vascular Biology and Translational Medicine, School of Medicine, Hunan University of Chinese Medicine, Changsha 410208, China; CAS Key Laboratory of Tropical Plant Resources and Sustainable Use, Yunnan Key Laboratory of Crop Wild Relatives Omics, Xishuangbanna Tropical Botanical Garden, Chinese Academy of Sciences, Kunming 650223, China

**Keywords:** CRISPR/Cas9, machine learning, lncRNA, sgRNA

## Abstract

CRISPR/Cas9 is a promising RNA-guided genome editing technology, which consists of a Cas9 nuclease and a single-guide RNA (sgRNA). So far, a number of sgRNA prediction softwares have been developed. However, they were usually designed for protein-coding genes without considering that long non-coding RNA (lncRNA) genes may have different characteristics. In this study, we first evaluated the performances of a series of known sgRNA-designing tools in the context of both coding and non-coding datasets. Meanwhile, we analyzed the underpinnings of their varied performances on the sgRNA’s specificity for lncRNA including nucleic acid sequence, genome location and editing mechanism preference. Furthermore, we introduce a support vector machine-based machine learning algorithm named CRISPRlnc, which aims to model both CRISPR knock-out (CRISPRko) and CRISPR inhibition (CRISPRi) mechanisms to predict the on-target activity of targets. CRISPRlnc combined the paired-sgRNA design and off-target analysis to achieve one-stop design of CRISPR/Cas9 sgRNAs for non-coding genes. Performance comparison on multiple datasets showed that CRISPRlnc was far superior to existing methods for both CRISPRko and CRISPRi mechanisms during the lncRNA-specific sgRNA design. To maximize the availability of CRISPRlnc, we developed a web server (http://predict.crisprlnc.cc) and made it available for download on GitHub.

## INTRODUCTION

Long non-coding RNAs (lncRNAs) are non-protein-coding transcripts longer than 200 nt [1]. With the wide application of next-generation sequencing, a large number of lncRNAs have been found in fungi, plants and animals [2, 3], and have been proven to play important and diverse functions in different important cellular processes [4–9]. Compared with protein-coding genes, the mechanisms by which lncRNAs exercise their functions are extremely complex and versatile [10]. In particular, many lncRNAs are confined to the nucleus, and the transcription of some lncRNAs may affect their target genes [11]. Therefore, the use of the CRISPR/Cas9 system, a revolutionary gene editing tool with cis-regulatory functions at the DNA level in cell nucleus, has tremendous advantages for lncRNA function study. Currently, CRISPR/Cas9 is commonly used to assay lncRNA functions [12–15], and even high-throughput functional screening of lncRNAs has been embarked on exploiting this technology [16].

CRISPR/Cas9 has become the most popular gene editing technology today, and different CRISPR/Cas9 derivative tools have been developed [17–39]. They can be divided into two categories depending on whether the Cas9 nuclease used is activated or not. CRISPR knock-out (CRISPRko) and knock-in (CRISPRki) methods use active Cas enzymes [40–45], where CRISPRko utilizes non-homologous end joining mechanism to cause gene knockout [46, 47], while CRISPRki utilizes homology-directed repair mechanism to insert a segment of DNA into the editing site [48, 49]. On the other hand, both CRISPR inhibition (CRISPRi) and activation (CRISPRa) methods use catalytically inactivated Cas9 (dCas9), which cannot cleave DNA but retains the ability of specifically bind to DNA [40, 50, 51]. When recruited to the promoter region, dCas9 can activate or inhibit transcription by either having an activator/repressor domain fused to it or by recruitment of an activator/repressor [52, 53]. Of these different techniques mentioned above, the most commonly used are CRISPRko and CRISPRi. Among them, CRISPRko is most used in gene editing targeting protein-coding gene, while CRISPRi is most used in lncRNA [54, 55]. The reason for this difference may be that lncRNAs do not have open reading frames, and CRISPRko on them maybe not as effective as CRISPRi. Therefore, designing paired sgRNAs to knockout large genome fragments may be a better strategy for CIRSPRko-based gene editing against lncRNAs [40]. Paired sgRNA design refers to the design of two sgRNA sequences located in two adjacent regions of the same target gene in CRISPR gene editing experiments. The purpose of this design is to achieve a double-stranded cleavage that introduces two sites at the same time in a specific region of the target gene, thereby inducing a large deletion of bases and generating a more significant knockout effect. CRISPETa is the first bioinformatics pipeline developed specifically for designing paired sgRNAs [56]. The results of using CRISPETa design in human cells have shown that predicted pairs of sgRNAs produce the expected deletions at high efficiency.

CRISPR/Cas9 gene targeting needs a custom single-guide RNA (sgRNA), which is crucial to improve the sensitivity and specificity of gene editing. Researches have shown that the guide sequence’s nucleotide composition is one of the most important determinants of sgRNA on-target efficiency [36, 57, 58]. In addition, many studies have confirmed the importance of GC content for sgRNA on-target activity [36, 41, 57, 59]. So far, a large number of sgRNA-designing software have been developed, which can be roughly divided into three categories. The first alignment-based approach is relatively simple [20, 22–25]. Basically, it only scans the PAM motif recognized by Cas9 and locates proximity sequences, as well as considers their potential off-target effect. The second category, hypothesis-driven tools, more complex in design [20, 26–31, 33–35, 58]. They score and rank sgRNAs based on specific features, such as GC content in sequences and RNA secondary structure. The last category is machine learning-based methods [17–19, 36–39, 57, 60]. These tools utilize machine learning methods, and appropriate datasets to train out models as a way to predict efficient sgRNAs. In recent years, due to the rapid development of deep learning techniques, neural network-based technology has been widely used and has achieved good results [61].

It is important to note that the above explorations are based on protein-coding gene datasets. Considering the significant differences between lncRNAs and protein-coding genes, it is necessary to investigate sgRNA-designing method optimized for lncRNAs. In our previous work, we constructed a manually curated database of validated sgRNAs for lncRNAs [55]. Based on these data and other data collected from literature, we evaluated and compared the performance differences of a series of known sgRNA-designing tools under coding and non-coding datasets. Our evaluation showed that the performance of most tools on lncRNAs is much lower than protein-coding genes. The performance was degraded on non-coding_CRISPRko dataset, and even worse on CRISPRi dataset. We analyzed the basis of these differences and found that, as compared with protein-coding genes, lncRNA-specific sgRNAs were significantly different in nucleic acid sequence, genome location and editing mechanism preference. Further, we proposed a new machine learning method, CRISPRlnc, for designing lncRNA-specific sgRNA in CRISPR/Cas9 system. Performance comparison shows that CRISPRlnc is far superior to existing methods for lncRNA-specific sgRNA design in both CRISPRko and CRISPRi mechanisms. To facilitate the use of CRISPRlnc, we developed a web server (http://predict.crisprlnc.cc) and made it available for download on GitHub. For the convenience of users, we integrate services such as paired sgRNA design and off-target risk analysis into the implementation of the CRISPRlnc tool, and provide a variety of information such as on-target validity, off-target risk and genomic location to help further select sgRNAs.

## MATERIALS AND METHODS

**Figure 1:**
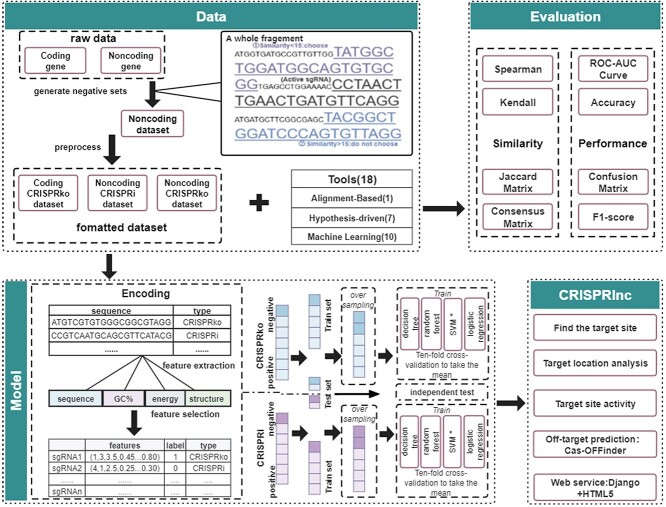
A complete workflow for the lncRNA-specific CRISPR/Cas9 sgRNA design.

As shown in Figure 1, our research workflow mainly includes four parts: data collection and processing, performance evaluation of existing sgRNA-designing tools, feature engineering and model construction, as well as CRISPRlnc software development and web service construction. The data and methods used are described in detail below.

### CRISPR/Cas9 sgRNA data collection and processing

In total, we used three sgRNA datasets of coding genes and three sgRNA datasets of non-coding genes (Table 1).

**Table 1:** Datasets detail information

Dataset	Species	Code Type	Mechanism	Quantity (e/in-e)
KBM7	*Homo sapiens*	Coding	CRISPRko	731/438
Mesc	*Mus musculus*	Coding	CRISPRko	1064/234
Zebrafish	*Danio rerio*	Coding	CRISPRko	52/59
Huh7.5	*Homo sapiens*	NonCoding	CRISPRko	544/263
iPSC	*Homo sapiens*	NonCoding	CRISPRi	431/35
MCF7	*Homo sapiens*	NonCoding	CRISPRi	45/52

*Note*. The e in the quantity column means efficient, and in-e means inefficient.

For the coding dataset, “KBM7” was originally proposed by Wang *et al*. in the human KBM7 CML cell line, and was processed by Xu *et al*., including 731 efficient sgRNAs and 438 inefficient sgRNAs [36, 57]. “Mesc” was created in a mouse cell line, and after processing the dataset by Xu *et al*. [36], 830 effective sgRNAs and 234 inefficient sgRNAs were identified. The last dataset “zebrafish” [17] was extracted from the supplemental data of CRISPOR [62], and compiled by Konstantakos *et al*. [61].

Non-coding datasets were retrieved from the CRISPRlnc database [55]. The first dataset “Huh7.5” was designed to screen for lncRNAs that positively or negatively regulate human cancer cell growth based on CRISPRko mechanism [63]. The two remaining datasets, “iPSC” and “MCF7”, are both based on CRISPRi mechanism and from the published datasets by Liu *et al*. [64]. The two datasets contain 16,401 lncRNA loci in six transformed cell lines and seven distinct cell lines of human induced pluripotent stem cells.

Due to the absence of experimentally validated inefficient sgRNAs in the collected data, we created artificial inefficient sgRNAs for all non-coding datasets. We followed a strategy used to generate negative datasets of protein interactions and oncogenes [65–67]: the negative set is the one that remains after excluding the positive set and the data with similarity to the positive set from the complete data set. The sequence composition of nucleotides is the factor that most affects the targeting activity of sgRNAs [36, 41, 57–59]. Therefore, we exclude sgRNAs that have some sequence similarity with positive sgRNAs in the vicinity of positive sgRNAs, and the remaining is the negative set. We scanned each efficient sgRNA and its upstream and downstream 15-nt sequences, and output all subsequences of 20 nucleotides with the beginning of the PAM motif. Then, we calculated the similarity scores between the efficient sgRNA and all the subsequences using Smith–Waterman algorithm [68], and assume that the subsequence similar to the efficient sgRNA is also effective, while the subsequence not similar to it (with a similarity score below 15) is inefficient.

### Performance evaluation of existing RNA-designing tools

#### Eighteen existing sgRNA-designing tools

As shown in Table S1, our evaluation has collected a total of 18 sgRNA-designing tools. In these tools, some of them, such as CRISPR_GE and CRISPick, output the sgRNAs they think are effective or ineffective. Some tools rated the effectiveness of each sgRNA but did not provide any recommendations. For these tools, we normalized their effectiveness scores to the interval [0,1] using maximum-minimum normalization, and delineated sgRNAs with the normalized score greater than 0.5 as the recommended sgRNAs. Table S2 demonstrated the normalized scores of sgRNAs in the dataset on each tool.

#### Evaluation metrics to measure the accuracy of the sgRNA design results

To measure the accuracy of the sgRNA design results, we defined four sgRNA samples following the confusion matrix format:

- TP (True Positive) is defined as a sample where the prediction is an effective sgRNA and the validation is also an effective sgRNA.
- FP (False Positive) is defined as a sample where the prediction is an effective sgRNA but the validation is not an effective sgRNA.
- TN (True Negative) is defined as a sample where the prediction is not an effective sgRNA and the validation is not an effective sgRNA.
- FN (False Negative) is defined as a sample where the prediction is not an effective sgRNA but the validation is an effective sgRNA.

We then use Precision, Sensitivity (Recall), Accuracy and F1 score for evaluating the performance of each model under the three datasets. The detailed metrics are described in Supplementary Document S1.

#### Evaluation metrics to measure the similarity of sgRNA design results between different tools

To evaluate the similarity of prediction results between different tools, we used a total of four metrics: Spearman correlation coefficient, Kendall correlation coefficient, Consensus matrix and Jaccard matrix. For the prediction tools that give a specific score for each sgRNA, the Spearman correlation coefficient was used to measure their correlation. The Kendall correlation coefficient is used to test the consistency of all tools for the classification of efficient and inefficient sgRNAs. In addition to describing the similarity of each tool to each dataset, we also proposed two metrics to describe the similarity of each tool to a whole gene sequence for sgRNA design: consensus matrix [69] and Jaccard coefficient [70]. These four similarity metrics are interpreted in Supplementary Document S1 In detail. 

### Feature engineering and model construction

We refer to previous studies [36, 57, 58, 71], and also use the base composition of sgRNA as important features to identify effectiveness of sgRNAs. In terms of GC content, not only the GC content of the whole sgRNA was identified and used, but also the GC values of the proximal and distal sequences of the PAM sequence were referred [19]. The RNA secondary structure and energetic characteristics are important for sgRNA activity. In particular, D. Dewran Kocak *et al*. [72] demonstrated that the hairpin structure of an RNA can influence the specific binding of its sgRNA. So we focused on the fraction bound as a stem, the hairpin structure, and the length of the free single-strand RNA. As a result, we extracted a total of 27 features, such as GC content, thermodynamic characteristics and secondary structure (Table S4 for more details). Furthermore, we computed the information gain of each feature and ranked them by XGBoost [73]. The main idea is to identify the optimal feature subset by calculating the accuracy of each subset, aiming to maximize the consistency of sgRNA validity within the same set after division. We introduced the features into the machine learning models one by one, starting from the feature with the largest information gain, and calculated the corresponding F1 scores until all features were gradually introduced. Finally, we selected 16 and 18 features for the sgRNA activity prediction models of the CRISPRko and CRISPRi mechanisms, respectively (see Figure S1 for more details).

In terms of model construction and training, due to the imbalance in the number of positive and negative samples in our collected non-coding sgRNA datasets, we first used SMOTE [74], a type of data augmentation technique that follows the basic idea of KNN for the minority class to over-sample the negative dataset. Further, given the small dataset of non-coding genes available for model training, complex models such as deep learning are likely to lead to model overfitting. Therefore, we chose to use relatively simple machine learning models, including logistic regression, decision tree, random forest and SVM, to build sgRNA on-target activity prediction models for non-coding genes. Considering the difference in the two mechanisms, we established two different classification models for CRISPRko and CRISPRi. All models were trained with 10-fold cross-validation. The grid search with 10-fold cross-validation is used to determine the best parameter values. The final model for both classifiers are implemented using the SVM in the sklearn library, which is significantly superior to other models. To ensure fairness, we selected a portion of data from each of the two datasets NonCoding_CRISPRko and NonCoding_CRISPRi as independent test sets (see Table S5). They only used for the final evaluation of the tool performance and to compare the performance with other tools.

For detailed information on all algorithms, software, and statistical methods used for feature engineering and model construction, please refer to Supplementary Document S1.

### CRISPRlnc software development and web service construction

We developed a web server using the Django framework (http://predict.crisprlnc.cc) and a desktop version in Python (https://github.com/Mera676/CRISPRlnc). The desktop version supports the input of whole genome sequences. The online version supports batch input in the FASTA format and can provide services for nine genomes. In the design of sgRNA based on CRISPRko, we provided the function of paired sgRNA design where the paired sgRNAs work together to knock out large genome fragments will result in the loss of function of the entire lncRNA gene. In the design of sgRNA based on CRISPRi, we can get the promoter sequence automatically by user specified length of the promoter sequence, and design sgRNA to inhibit the transcription of lncRNA. We also provide the ability to design targets downstream of the gene, which only requires users to select the length of the downstream sequence. To help users further evaluate the performance of sgRNA, we have incorporated off-target risk analysis for each target into the implementation of the CRISPRlnc tool. We calculated off-target risk integrating mismatch types and mismatch locations based on the CFD score published by Doench *et al* [21]. In addition, by integrating the three scores for on-target validity, off-target risk and genomic location, we provide a composite weighted score for each sgRNA, with higher scores being better. The details of this composite score are described in Supplementary Document S1.

## RESULTS

### Performance evaluation of existing sgRNA-designing tools

**Figure 2:**
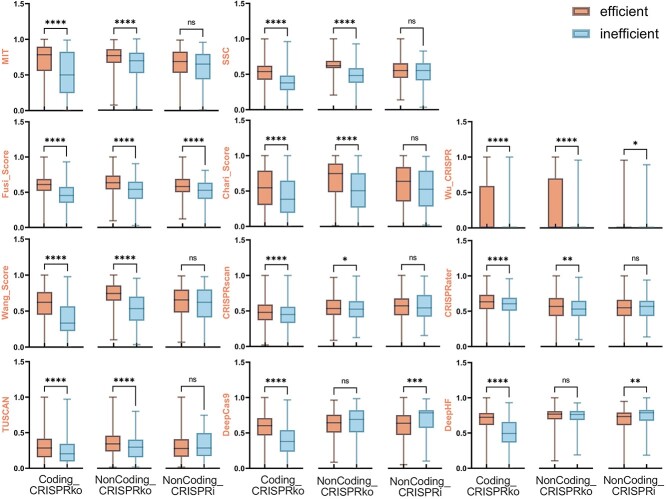
Comparison of 11 software scores of effective and ineffective sgRNAs on three different datasets. The vertical axis of each sub-graph indicates the normalized scores of each tool. The scores of effective and ineffective sgRNAs in different datasets were represented using boxplots, and statistical significance tests were conducted (*t*-test, ^*^P-value 0.05, ^*^^*^P-value 0.01, ^*^^*^^*^P-value 0.001, ^*^^*^^*^^*^P-value 0.0001).

The ideal scoring algorithm should reward positive samples with high scores and penalize negative samples with low scores. Among the 18 tools we evaluated, 11 tools (MIT, Chari_Score, Fusi_Score, SSC, Wu_CRISPR, Wang_Score, CRISPRscan, CRISPRater, TUSCAN, DeepCas9, DeepHF) rated the effectiveness of sgRNAs. Figure 2 shows how the positive and negative samples in the three datasets scored on each scoring algorithm. Each of these 11 algorithms performs well in the coding_CRISPRko dataset, and the scores of effective sgRNAs are significantly higher than that of ineffective sgRNAs. For lncRNA data, on the lncRNA CRISPRko dataset, there are four software—CRISPRater, CRISPRscan, DeepCas9 and DeepHF—that cannot ensure a good distinction between positive and negative samples; on the lncRNA CRISPRi dataset, the performance of all these software further decreased, with only Fusi_Score has the ability to discriminate between positive and negative sets, while all the other tools do not show good discrimination. Especially DeepCas9 and DeepHF, the scoring system of these two software is contrary to what we expected, and the scores on the inefficient sgRNAs turned out to be significantly higher than the sgRNAs.

**Figure 3:**
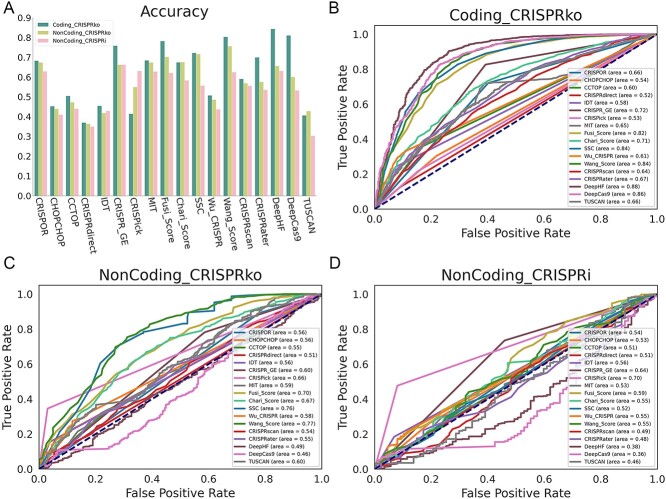
Performance evaluation of known sgRNA-designing tools on three different datasets. (**A**) is the Accuracy of all tools for dichotomous sgRNAs. (**B**)–(**D**) are the ROC curves of each tool under coding gene knock-out mechanism dataset, non-coding gene knock-out mechanism dataset and non-coding gene inhibition mechanism dataset, respectively.

We further conducted the performance evaluation of all these eighteen software (Detailed confusion matrix values and performance metrics for the tools are shown in Table S3). From Figure 3(A), we can see that, with a few exceptions, the prediction accuracy of most software shows a gradual downward trend, from Coding_CRISPRko to NonCoding_CRISPRko to NonCoding_CRISPRi. CRISPick is a significant exception, possibly because it considers sgRNA design of CRISPRi mechanism alone and took into account where the target sequence is within a known ENCODE annotated DNase I Hypersensitive Site. Further, ROC curves showed similar trends, with almost all software showing similar gradual declines in prediction performance (Figure 3B–D, see Figure S2 for more details). Another issue that caught our attention was that the tools DeepCas9 and DeepHF, which achieved the best results on the Coding_CRISPRko dataset, but performed the worst on the non-coding dataset, even with AUC below 0.5. We speculated that these two models, due to their strong ability in deep learning, captured rich details on the coding dataset, but these features were not suitable for the non-coding dataset, resulting in the most significant decrease in performance.

**Figure 4:**
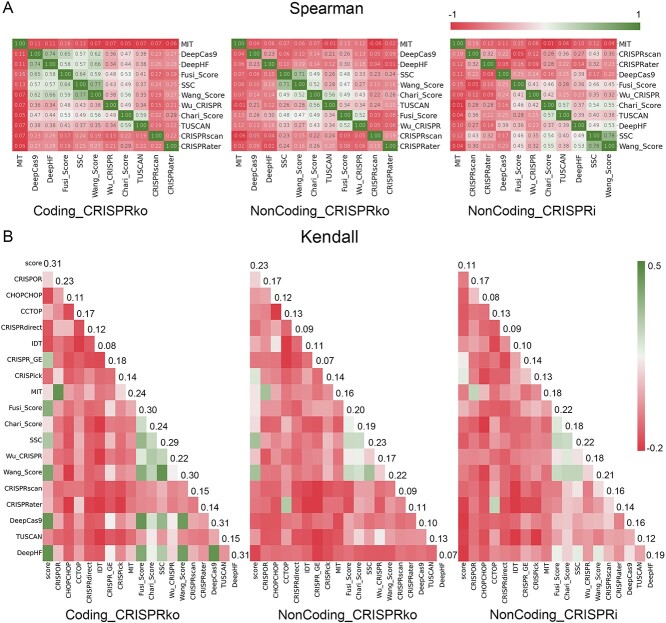
The difference in similarity between the predictions of known sgRNA-designing tools on three different datasets, with green indicating good similarity and red indicating poor similarity. (**A**) is the Spearman similarity on the three datasets, and numbers in the cells correspond to the Spearman similarity scores. (**B**) is the Kendall similarity on the three datasets, and the score’ column is the Kendall similarity score between each prediction tool and the original classification of sgRNA in each dataset. The number marked after each row refers to the average Kendall similarity of the tool’s classification with other tools.

We also compared the prediction consistency of each tool in different datasets using Spearman correlation (Figure 4A). We found that the correlations between scoring tools varied across datasets. On Coding_CRISPRko dataset, there are five tools—DeepCas9, DeepHF, Fusi_Score, SSC and Wang_Score—that show a strong scoring correlation between each pair of them (correlation coefficient > 0.5). Conversely, there are few tools that show a strong scoring correlation on non-coding datasets (Table S6 for more details). We then used the Kendall correlation coefficient to assess the concordance of the classification results of each tool for both effective and ineffective sgRNAs (Figure 4B). In good agreement with Spearman analysis, these tools have significantly better classification synchronicity on the coding dataset compared to the non-coding dataset. It is worth mentioning that by calculating the mean Kendall coefficient of each tool and the original classification labels of the three datasets, we found that the classification results on Coding_CRISPRko dataset was the closest to the true value, with a correlation coefficient of 0.31. The next closest was NonCoding_CRISPRko (0.23), and the worst was NonCoding_CRISPRi (0.11) (Table S7 for more details).

In addition, we compared the consistency of prediction results when different sgRNA design tools were applied to long gene sequences. We selected 12 tools that can scan long gene sequences for sgRNA design and compared the consistency of their prediction results. In Figure S3, the consensus matrix and Jaccard clustering of the 12 tools under coding genes (*TP53*) and non-coding genes (*HEIH*) are shown. In the non-coding gene *HEIH*, the prediction results of five tools (CRISPOR, CRISPick, CRISPR_GE, CHOPCHOP, DeepHF ) cover most of the prediction results of other tools. In the coding gene *TP53*, the prediction results of SSC also showed high coverage in addition to the five tools aforementioned. In terms of similarity of prediction results, the tools clustered into two clusters on non-coding genes, while on coding genes, the predictions among tools aggregated into a broader large cluster. This indicates that the tools had higher uniformity of design results on coding genes, but the tools did not reach a uniform standard when designing sgRNAs on non-coding genes.

### Distinct preferences of sgRNA towards different sequence and structure features across the three datasets

**Figure 5:**
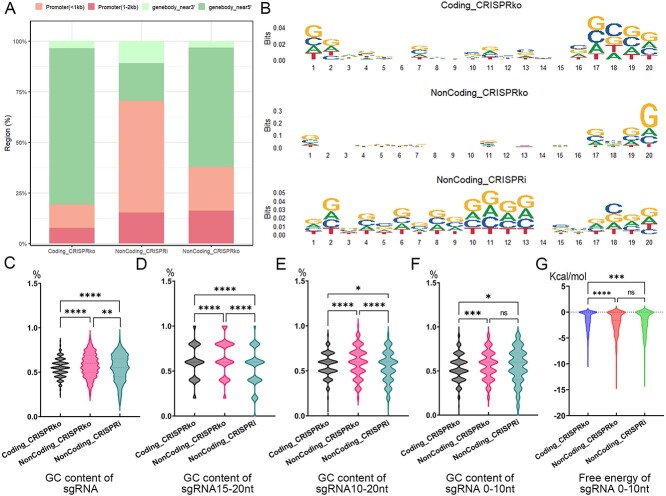
Distinct preferences of sgRNA towards different sequence and structure features across the three datasets. (**A**) is a comparison of the genomic localization of sgRNAs in the three datasets. (**B**) is the possibility of base distribution at each position of sgRNA sequences in the three datasets. (**C**)–(**F**) is the GC content distribution of each region of sgRNA sequences in the three datasets (*t*-test, ^*^P-value 0.05, ^*^^*^P-value 0.01, ^*^^*^^*^P-value 0.001, ^*^^*^^*^^*^P-value 0.0001). (**G**) shows distribution of the minimum free energy required to unravel the secondary structure of sgRNA in the three datasets (*t*-test, ^*^P-value 0.05, ^*^^*^P-value 0.01, ^*^^*^^*^P-value 0.001, ^*^^*^^*^^*^P-value 0.0001).

 Previous studies have shown that the efficiency of Cas9 cleavage primarily depends on the base preference at particular positions and the GC content of the guide RNA sequence, which conducted the on-target activities of predicted sgRNAs [19, 36]. In addition, the secondary structure, energetic features and fragment GC content of sgRNAs can also have an impact on cleavage effectiveness [19]. Therefore, we focused on studying the different preferences of these features under the three datasets (Figure 5). We used *t*-tests to compare the significance of differences in feature preferences under the three datasets.

We first compared the genomic localization of sgRNAs in the three datasets (Figure 5A). The sgRNAs on the Coding_CRISPRko dataset are mostly distributed in the gene body region near the 5’ end. In contrast, the sgRNAs on the NonCoding_CRISPRi dataset show a distinct preference to the promoter region, especially the core promoter region. The genomic distribution of sgRNAs on the NonCoding_CRISPRko dataset is somewhere in between, with the highest portion resided in the proximal 5’ end of gene body region, and the proportion of those located in the promoter region increased significantly as well. The different preferences of genome localization also lead to differences of the base distribution at each position of sgRNA sequences on the three datasets (Figure 5B). In NonCoding_CRISPRko dataset, sgRNAs show very pronounced guanine enrichment at the twentieth position of the sequence (Figure 5B). While sgRNAs on the NonCoding_CRISPRi dataset exhibit a strong guanine preference in the middle of the sequence. We also compared the differences of GC content in sgRNA sequences on the three datasets in detail (Figure 5C–F). We can see that the sgRNA from different datasets have different GC content, especially the GC content in the tail (15–20 nt) of the sequence is significantly different. Changes in the GC content of RNA sequences may affect their stability and folding energy. Thus we further compared the distribution of the minimum free energy required to unravel the possible variation of secondary structure of sgRNAs in the three datasets, and the results showed that there were significant differences in the distribution of the minimum free energy between the sgRNAs in the coding dataset and the sgRNAs in the other two sets (Figure 5G).

### Constructing a machine learning method for lncRNA-specific sgRNA design

**Figure 6:**
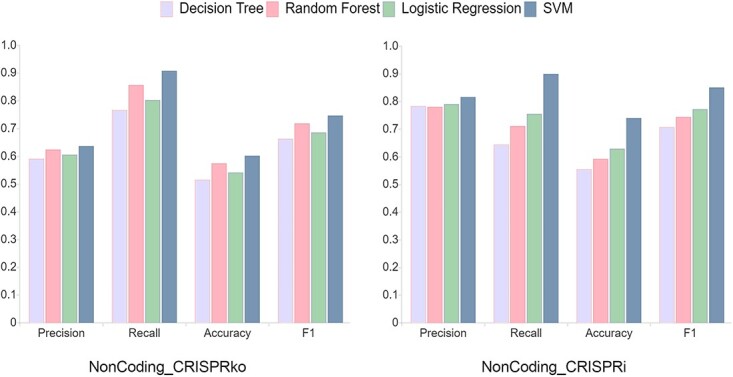
10-fold cross-validation scores of four models (decision tree, random forest, logistic regression and SVM) under NonCoding_CRISPRko and NonCoding_CRISPRi training sets.

From the above analysis, it can be seen that the sgRNA design for non-coding genes not only has its own unique features compared to coding genes, but there are also many differences between the two mechanisms - CRISPRko and CRISPRi—for non-coding genes themselves. Therefore, we need to construct machine learning models for these two mechanisms separately. In Figure 6, we constructed and compared the performance of four models based on different algorithms, including decision tree, random forest, logistic regression and SVM. All models were trained on two datasets—NonCoding_ CRISPRko and NonCoding_CRISPRi—by 10-fold cross-validation and the global best parameters were selected in the grid search. The results showed that SVM was significantly superior to other models on both the NonCoding_CRISPRko and NonCoding_CRISPRi datasets. Therefore, we used SVM as the basis for the further optimization

**Figure 7:**
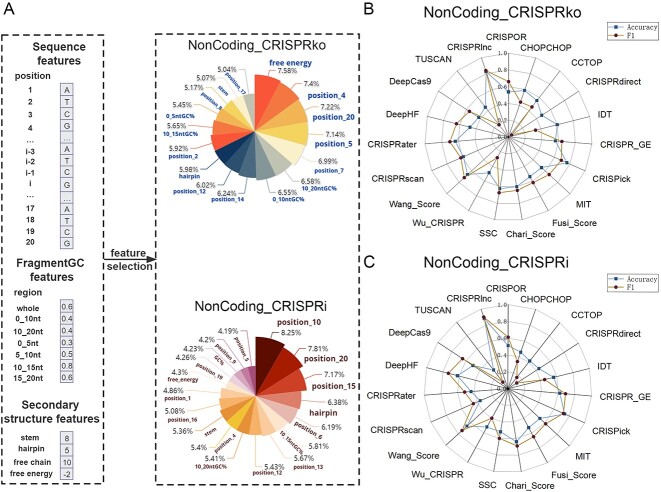
Feature selection results and performance comparison results of the two models. (**A**) is the feature importance on the two datasets; in NonCoding_CRISPRko, we extracted 16 best features, and in NonCoding_CRISPRi, we extracted the 18 best features from all 27 features. (**B**) is the performance comparison of CRISPRlnc with other tools under the independent NonCoding_CRISPRko test dataset for both Accuracy and F1-score metrics. (**C**) is the performance comparison of CRISPRlnc with other tools under the independent NonCoding_CRISPRi test dataset for both Accuracy and F1-score metrics.

Considering the quantitative imbalance between the positive and negative samples in the sgRNA datasets, we used SMOTE to oversample the negative dataset. From Table 2, it can be seen that the overall performance of the model has been significantly improved after SMOTE processing. It should be noted that the Recall rates in both datasets have decreased. This is because the training set had much more positive samples than negative samples before oversampling. Therefore, the model can predict that most samples are positive, resulting in a falsely high Recall rate. After oversampling, the data of the positive and negative sets tend to be balanced, and the Recall rate reflects the real situation of the model. Next, we tried to reduce the overfitting risk of the model by selecting limited features while ensuring the model’s tenfold cross-validation performance. First, we ranked the features using information gain for each feature that affects sgRNA cleavage activity. Then, we sequentially introduced the sorted features into the SVM model and checked the performance of the model. We observed that under the CRISPRko mechanism, the model performance was optimized when 16 features were selected. Correspondingly, under the CRISPRi mechanism, the optimal model performance occurs when 18 features are introduced. The results showed that the reduction of features used in the model did not result in a decrease in its performance (Table 2). In addition, the results of feature selection also show that the sgRNA design for lncRNA is very different against CRISPRko and CRISPRi mechanisms (Figure 7A). The characteristic of lower free energy, which leads to better binding strength, is crucial for the design of lncRNA-specific sgRNA for both CRISPRko and CRISPRi mechanisms. However, in the CRISPRko mechanism, this feature ranks first, which is much higher than in CRISPRi mechanism. On the other hand, for the CRISPRi mechanism, the tenth base composition of sgRNA has the highest information gain and ranked first in importance among the selected features. In addition, we have found a very pronounced enrichment of terminal guanines in the NonCoding_CRISPRko effective sgRNA collection (Figure 5B), while this feature is also captured by the model and serves as an effective discriminator between effective and ineffective sgRNAs.

**Table 2:** SVM model evaluation after SMOTE oversampling and feature selection under training sets. (10-fold cross-validation scores)

Dataset	Model	Precision	Recall	Accuracy	F1
NonCoding_CRISPRko	SVM	0.6372	0.9085	0.6026	0.7474
	Optimized SVM	0.8042	0.7877	0.8015	0.7879
	Further optimized SVM	0.878	0.8431	0.8119	0.8081
NonCoding_CRISPRi	SVM	0.8163	0.8889	0.7407	0.8511
	Optimized SVM	0.9353	0.8102	0.8754	0.8648
	Further optimized SVM	0.9556	0.8218	0.8865	0.8794

*Note*. The optimized SVM means the SVM model after SMOTE, and the further optimized SVM means the SVM model after SMOTE and feature selection.

Furthermore, we found that the model performance on the independent test set also improved after the feature selection (Table 3). After feature selection, the average Precision, Accuracy and F1 score on the NonCoding_CRISPRko independent test set have increased from 82.5, 80 and 80.95% to 90, 82.5 and 83.72%. On the NonCoding_CRISPRi independent test set, these numbers have increased from 86.67, 80 and 82.76% to 90, 88 and 90%. Using independent datasets, we also compared the performance of our optimized model with existing sgRNA-designing tools. The results showed that the CRISPRlnc model performs much better than existing sgRNA-designing tools on both NonCoding_CRISPRko and NonCoding_CRISPRi independent test sets. The CRISPRlnc model’s Accuracy and F1 score on the NonCoding_CRISPRko dataset are 80 and 80.48%, respectively (Figure 7B); on the NonCoding_CRISPRi dataset, the CRISPRlnc model’s Accuracy and F1 score are 88 and 90%, respectively (Figure 7C). To evaluate our tool more comprehensively, we collected and organized 36 sgRNA pairs on 11 genes from 10 papers, and Table S9 details the information of these sgRNAs as well as the prediction scores of CRISPRlnc. The results show that CRISPRlnc predicted 26 of the 36 sgRNA pairs. So, we have reason to believe that CRISPRlnc will be a good tool for CRISPR/Cas9 paired-sgRNA design.

**Table 3:** Performance changes of CRISPRlnc before and after feature selection on independent test sets

Dataset	State	Precision	Recall	Accuracy	F1
NonCoding_CRISPRko	Before feature selection	0.8250	0.7500	0.7750	0.7857
	After feature selection	0.8250	0.7857	0.8000	0.8048
NonCoding_CRISPRi	Before feature selection	0.7719	0.8846	0.8000	0.8215
	After feature selection	0.9000	0.9000	0.8800	0.9000

### CRISPRlnc software and web service

**Figure 8:**
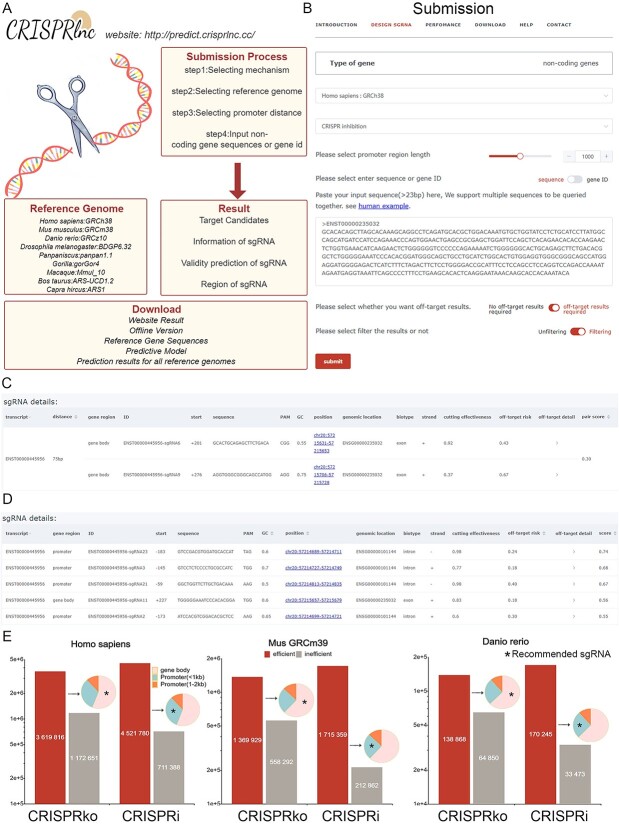
Overview of CRISPRlnc web version. (**A**) Services and downloads available on the website. (**B**) Examples of the website usage. (**C**) sgRNA design results based on CRISPRko mechanism. (**D**) sgRNA design results based on CRISPRi mechanism. (**E**) Statistics of sgRNA target results for lncRNA from *Homo sapiens*, *Mus musculus* and *Danio rerio*.

Based on the optimized model, we have constructed an online version of CRISPRlnc (http://predict.crisprlnc.cc/), and also provided CRISPRlnc program download on GitHub (https://github.com/Mera676/CRISPRlnc). As shown in Figure 8(A, B), the website provides easy access to services and concise interface. The output results of CRISPRlnc include the sequence, genome localization, on-target activity score and off-target risk of each candidate sgRNA. When designing sgRNAs for lncRNA genes, in addition to considering the cutting effectiveness of the target, we also need to consider specific off-target situations and genome localization. Therefore, by integrating the three scores for on-target validity, off-target risk and genomic location, we provide a composite weighted score between [−1,1] for each sgRNA, with higher scores being better. The output of CRISPRlnc is slightly different for CRISPRko and CRISPRi mechanisms (Figure 8C, D). We designed single sgRNA for the CRISPRi mechanism with higher scores for targets located in the gene promoter region, and paired sgRNAs for the CRISPRko mechanism, with higher scores for paired targets located in the lncRNA gene body region and with appropriate distance between two sgRNAs. Furthermore, we support designing sgRNAs by Ensemble id or gene symbol, and can design against all the transcript variants of one gene at the same time. In addition, the tool can automatically retrieve promoter sequences and design sgRNAs against them, and can also be user-specified for promoter sequence length. The website provides the results of large-scale sgRNA prediction using CRISPRlnc for human, mouse and zebrafish lncRNAs (Figure 8E). The predictions included all cleavage-effective sgRNAs under the CRISPRko and CRISPRi mechanisms. Furthermore, based on the genomic location of the effective sgRNAs, we screened for the recommended sgRNAs. On average, each lncRNA has approximately 50 recommended sgRNAs. Among them, human lncRNA corresponds to the most recommended sgRNAs, with about 60 and 100 recommended sgRNAs for CRISPRko and CRISPRi mechanisms, respectively (Table S8 for more details).

## DISCUSSION

LncRNAs are very different from protein-coding genes in terms of genome localization, base composition and the ways they function. All these characteristics determine that we need to consider the sgRNA design of lncRNA separately [40, 54]. In the analysis of target validity, we identified unique sequence and structural features of lncRNA-specific sgRNA design that differ from protein-coding genes. More importantly, we have developed a software package and web service for lncRNA-specific sgRNA design for the first time, providing practical solutions for researchers interested in using CRISPR/Cas9 for lncRNA functional research. We believe that our work is just the beginning, which will inspire more researches in this area and enable a better understanding of the function of lncRNAs.

CRISPR/Cas9 technology has been widely used in various fields with successful applications [75]. However, designing sgRNA remains a challenge. A large amount of research has been conducted on how to design sgRNA, but they are all based on protein-coding genes. In this work, we found that these software designed based on protein-coding gene data did not work well in lncRNA data, especially in Noncoding_CRISPRi dataset. Considering that the CRISPRi system often targets promoter regions to interfere with gene function without introducing DNA cleavage [51, 52], this is expected. But somewhat surprisingly, the two deep-learning-based methods that with the best performance in protein-coding gene data had the worst performance in lncRNA data. We suspect that this is because these two deep learning methods have the deepest insight into the details of the features of protein-coding genes. However, these feature patterns are likely not served for lncRNA data, resulting in the fastest degradation of their performance. On the other hand, our CRISPRlnc model introduces a lot of lncRNA-specific features. For example, effective sgRNAs in NonCoding_CRISPRko dataset show significant enrichment of guanine at the twentieth position of the sequence, and there are significant differences in the distribution of the minimum free energy required to unravel the possible variation of secondary structure of sgRNAs between coding and non-coding datasets. The introduction of these lncRNA-specific features is an important reason why our CRISPRlnc model outperforms the existing models based on protein-coding gene datasets.

Unlike coding genes, lncRNAs do not have open reading frames, and small INDELs are likely not to affect their expression and function. Therefore, two approaches are adopted for efficient CRISPR gene editing of lncRNAs. The first is based on the CIRSPRi mechanism, which selects to design sgRNAs in promoter regions of lncRNA genes, so that a single sgRNA may affect gene transcription; the other is based on the CIRSPRko mechanism, which selects to design sgRNAs in lncRNAs’ gene body regions and usually requires the design of paired sgRNAs. Simultaneous operation of paired sgRNAs can be used for the knockout of large fragment of gene body region, which will result in the loss of function of the whole lncRNA gene. For this reason, we trained and constructed independent prediction models for CRISPRko and CRISPRi mechanisms to design lncRNA-specific sgRNAs for each mechanism. In addition, we have also provided single sgRNA design in lncRNA gene promoter region for CIRSPRi mechanism and paired sgRNA design in gene body region for CIRSPRko mechanism on the CRISPRlnc website. Another issue that needs to be noted in the design of lncRNA-specific sgRNA is that in many cases, lncRNA genes will overlap with coding genes on the genome. For this reason, we have provided genomic location information of sgRNAs on the CRISPRlnc website to help users more easily find this kind of overlap and prevent the designed sgRNA from affecting other non-target genes. All these unique designs will greatly facilitate the design of lncRNA-specific sgRNAs.

There has been a significant amount of work focused on designing sgRNAs for protein-coding genes. These efforts have not only produced a large number of sgRNA-designing tools, but also brought a wealth of experimental data for sgRNA performance testing. For specific coding genes, the effectiveness of various sgRNA sequences has been meticulously evaluated, including both effective and ineffective ones. In contrast, research on sgRNAs targeting lncRNAs is still in its early stages. Through literature search, we collected a lot of effective sgRNA data on lncRNA. However, there is still little work on large-scale performance evaluation of lncRNA specific sgRNA, and the lncRNA datasets that we have used do not have information on ineffective sgRNAs. The difficulty of finding experimentally validated negative datasets has also occurred in other research fields. For example, it is easy to collect experimentally validated cancer-related lncRNA data, but it is difficult to collect experimentally validated cancer-unrelated lncRNA data. Another example is protein–protein interaction, where there is a large number of experimentally validated protein interaction data, but few reported experimentally validated non-interacting protein data. In machine learning research in these areas, researchers have come up with a strategy for constructing negative datasets, which has provided us with inspiration to create artificial inefficient sgRNAs [65–67]. However, we are uncertain how closely these artificial sgRNAs mimic real-world inefficient sgRNAs in terms of sequence composition and functional ineffectiveness, and creating artificial inefficient sgRNAs for non-coding datasets can only be a stopgap measure. We believe that in the current situation, CRISPRlnc will provide valuable assistance to researchers in sgRNA design of lncRNAs. In the future, with the continuous deepening of research in CRISPR/Cas9 on lncRNAs, more large-scale evaluation experiments of lncRNA-specific sgRNA performance will be brought to optimize the lncRNA-specific sgRNA design tools.

Although there is some general knowledge about the effectiveness of sgRNAs, there are still some variations in different species and cell lines [36, 41, 57, 59].Recent studies have shown that the performance of sgRNA in genome editing may vary depending on the cell type used [76]. Differences in chromatin structure, changes in the expression level and location of various nuclease, and variations in the expression level of genes involved in DNA damage response and cell cycle regulation may affect the efficiency and specificity of CRISPR/Cas9 system in different cell types [36, 58, 77, 78]. Therefore, the selection of datasets may have issues with representation and potential biases. Whether the datasets used to train sgRNA-designing machine learning models fully represents different cell types, organisms and lncRNA characteristics will be an important factor that may affect the generalizability of the model. In our current work, we have made every effort to collect data from different species and cell lines, but it must be acknowledged that the large-scale experimental data on lncRNA-specific sgRNA design is still insufficient. The inclusion of a broader range of datasets in the future will benefit the further work on lncRNA-specific sgRNA design. In the future, with the increase of lncRNA-specific sgRNA data from different species and cell lines, more features may be added to the model, such as epigenetic modifications of the genome or tissue-specific gene expression [79–81]. all of which will bring significant optimization for the subsequent improvement of CRISPRlnc.

Another point to consider is the principles of sgRNA design to maximize on-target activity and minimize off-target risk [82, 83]. Off-target risk is a major factor affecting sgRNA performance. Previous studies have shown that the degree of sequence similarity as well as the location where the mismatch occurs are decisive factors influencing the off-target effect [21, 82, 84–86]. Hsu *et al*. [82] and Doench *et al*. [21] have precisely summarized the possibility of calculating the genome-wide off-target risk of sequences based on the mismatch type, mismatch location, and other factors under the auspices of batch experiments, and the two algorithms are also currently used by a large number of mainstream design tools. Although there are some dataset differences, there is a certain generalization about the off-target risk for both coding and non-coding genes: the lower the number of mismatches or the closer the mismatch location is to the distal end of the PAM will result in a greater off-target risk. At present, there are no off-target risk studies for lncRNA-specific sgRNAs, and our work also mainly focused on sgRNA on-target activity. With the increase of large-scale evaluation experimental data on the performance of lncRNA-specific sgRNA, we should conduct specific analysis on the off-target risk of these sgRNAs and search for possible lncRNA-specific features. Based on a large number of reliable non-coding CRISPR/Cas9 sgRNA off-target experiments, fine-tuning the traditional off target risk algorithms is a challenge and opportunity for non-coding gene off-target risk analysis.

**Key Points**

- Machine learning algorithm built for lncRNA-specific sgRNA design of CRISPR/Cas9 system based on non-coding RNA characteristics.
- Compensating for the lack of performance of existing tools on non-coding datasets and supporting the design of sgRNAs against both CRISPRko and CRISPRi mechanisms.
- Launching a user-friendly web server (http://predict.crisprlnc.cc), with paired sgRNA or single sgRNA design service and off-target risk analysis.

## Supplementary Material

Document_S1_detailed_description_of_the_formula_theorem_bbae066

FigureS1_bbae066

FigureS2_bbae066

FigureS3_bbae066

Table_S1_The_detailed_information_of_all_tools_bbae066

Table_S2_Formatted_datasets_and_regularization_scores_of_bbae066

Table_S3_Confusion_matrix_and_F1_score_for_all_tools_bbae066

Table_S4_Feature_details_and_information_gain_values_bbae066

Table_S5_CRISPRlnc_train_datasets_and_test_datasets_bbae066

Table_S6_Spearman_coefficients_for_all_tools_on_the_three_datasets_bbae066

Table_S7_Kendall_coefficients_for_all_tools_on_the_three_datasets_bbae066

Table_S8_Statistics_of_sgRNA_prediction_Danio_rerio_bbae066

Table_S9_paired-sgRNA_information_and_CRISPRlnc_prediction-results_bbae066

## DATA ACCESSIBILITY

All the processed data and the source code of CRISPRlnc and all the comparative algorithms are available at GitHub: https://github.com/Mera676/CRISPRlnc. All data used to evaluate the tools and train the predictive models are available in Tables S1, S2 and S5.
