# Supplementary material for: CRISPRlnc: a machine learning method for lncRNA-specific single-guide RNA design of CRISPR/Cas9 system
Source: Brief Bioinform. 2024 Feb 29;25(2):bbae066. doi: 10.1093/bib/bbae066 (PMC10905519; doi:10.1093/bib/bbae066)
Supplement: Document_S1_detailed_description_of_the_formula_theorem_bbae066 [file document_s1_detailed_description_of_the_formula_theorem_bbae066.docx]

**Detailed explanation of the formulas and theorems**

**1. Evaluation metrics to measure the accuracy of the sgRNA design results**

**1) Precision** indicates the probability that the prediction is indeed effective sgRNA among all the samples predicted to be effective sgRNA, which is used to measure the accuracy of the tool's prediction, its value is calculated using the following formula:

$$\begin{aligned} Precision=\frac{\mathrm{TP}}{TP+FP}\#\left( 1 \right) \end{aligned}$$

**2) Sensitivity** of the tool is measured by using Recall to indicate the probability that an effective sgRNA is correctly predicted. Similar to Precision, a higher value indicates a better performance of the tool. and its value is calculated using the following formula:

$$\begin{aligned} Recall=\frac{\mathrm{TP}}{TP+FN}\#\left( 2 \right) \end{aligned}$$

**3) Accuracy** is a more comprehensive metric to indicate the probability of positive and negative sgRNAs being successfully classified, its value is calculated using the following formula:

$$\begin{aligned} Accuracy=\frac{TP+TN}{TP+TN+FP+FN}\#\left( 3 \right) \end{aligned}$$

**4) F1 Score** is a statistical measure of the accuracy of a binary classification model. F1 score can be considered as a summed average of the precision and recall of the model, and is calculated by the following formula:

$$\begin{aligned} F1=\frac{2*P r e c i s i o n*R e c a l l}{Precision+Recall}=\frac{2*T P}{2*T P+FP+FN}\#\left( 4 \right) \end{aligned}$$

**5) ROC curve:** The full name is receiver operating characteristic curve, which is a composite indicator reflecting the sensitivity and specificity of continuous variables. **AUC** is calculated as the Area Under the ROC Curve. Larger AUC indicates higher accuracy of the tool for sgRNA classification.

**2. Evaluation metrics to measure the similarity of sgRNA design results between different tools**

**1) Spearman coefficient:** The formula for the **Spearman coefficient** is shown below:

$$\begin{aligned} r_{s}=1-\frac{6\sum_{n}^{i=1} d_{i}^{2}}{n\left( n^{2}-1 \right)}\#\left( 5 \right) \end{aligned}$$

where *d_i_* is the pairwise difference between the ranks of the two variables *x_i_* and *y_i_*, and n represents the number of samples.

**2) Kendall’s correlation:** The formula for **Kendall’s correlation** coefficient is shown below:

$$\begin{aligned} \tau=\frac{2\left[ n_{c}-n_{d} \right]}{n\left( n-1 \right)}\#\left( 6 \right) \end{aligned}$$

where *n_c_* is the number of concordant pairs, and *n_d_* is the number of discordant pairs.

**3) Consensus matrix:** The **consensus matrix** aims at describing the similarity of design outcomes among tools ^[64]^, and the matrix’s formula is:

$$\begin{aligned} \left[ \begin{matrix} C_{11} & \cdots& C_{1n} \\ \vdots& C_{ij} & \vdots\\ C_{n1} & \cdots& C_{\mathrm{nn}} \end{matrix} \right]_{n*n}\#\left( 7 \right) \end{aligned}$$

where *C_i_*_j_ indicates the coverage of sgRNA predicted by tool *j* on tool *i.* For example, *C_13_* = 0.7 means that 70% of the sgRNAs predicted by tool 1 are also predicted by tool 3. And *C_31_*=0.7 means that 70% of the sgRNAs predicted by tool 3, are also predicted by tool 1. Note that *C_ij_* and *C_ji_* are not equal.

**4) Jaccard coefficient:** The **Jaccard** coefficients are used to additionally describe the similarity of each two tools’ results, defining the Jaccard coefficient ^[65]^ as:

$$\begin{aligned} J\left( I,J \right)=\frac{I\cap J}{I\cup J}\#\left( 8 \right) \end{aligned}$$

It represents the proportion of the number of elements of the intersection of the two sets I and J in the concatenation of I, J.

**3. Algorithms, software, and statistical method used in feature engineering and model construction**

We extracted a total of 27 features, such as GC content, thermodynamic characteristics, and secondary structure. The software used for feature extraction includes RNAeval (for thermodynamic features), and RNAfold (for secondary structure) in Vienna RNA Package.

For each feature, we used Student's t-test to compare the significance of differences in feature preferences under the three datasets, and then computed the information gain of each feature and ranked them by XGBoost. The software used for statistical testing is the t.test function in GraphPad Prism 9. The software used for calculating the information gain of each feature and for feature sorting includes XGBClassifier and SelectFromModel in sklearn library.

We used SMOTE, a type of data augmentation technique, to over-sample the negative dataset. The SMOTE implementation we used is the python package imbalanced-learn. We used logistic regression, decision tree, random forest, and SVM to build sgRNA on-target activity prediction models for noncoding genes. The specific machine learning models are constructed using the python package sklearn.svm, sklearn.RandomForestClassifier , sklearn.DecisionTreeClassifier, and sklearn LogisticRegression. We used a grid search with 10-fold cross-validation to determine the optimal parameter values for the machine learning models. The grid search we used is the python package sklearn.model_selection.GridSearchCV.

**4. The formula of sgRNA composite score (F)**

The **sgRNA composite score (*F*)** was quantified as [-1,1], with higher score indicating a better effect of the sgRNA. On-target validity (*S* _validity_) is a score quantified as [0,1], the closer the score is to 1, the better the on-target cleavage ability of the site. Off-target risk (*S* _risk_) is a score quantified as [0,1], the higher the score is, the higher is the risk of off-targeting of the site on a genome-wide basis. Genomic location (*S* _location_) is a discrete value used to distinguish the performance effect of designing targets on different gene regions (promoter, gene body, gene downstream). When designing a sgRNA, we want it to be located in a suitable gene region (CRISPRko mechanism prefers to design targets in gene body region, CRISPRi mechanism prefers to design targets in promoter region), and it also has high on-target validity and low off-target risk. Our collection of positive sgRNAs is experimentally validated targets. We converted each sgRNA to (*S* _location_, *S* _validity_, *S* _risk_) coordinates, mapped it to a 3D space, and de-fitted a 3D surface such that all positive sgRNAs are above that plane. The formula for this 3D surface is:

$$\begin{aligned} F=\tanh\left( \begin{aligned} 0.22S_{location}+0.33S_{validity}-0.13S_{risk}+0.31S_{location}S_{validity} \\ +0.114{S^{2}}_{validity}-1.21{S^{2}}_{risk}+0.14 \end{aligned} \right)\#\left( 9 \right) \end{aligned}$$
